# Excellent 2‐Year Outcomes of MUIB All‐Inside ACL Reconstruction: A Registry‐Based Analysis of 63 Patients With Zero Graft Failure

**DOI:** 10.1111/os.70442

**Published:** 2026-10-15

**Authors:** Badrul Akmal Hisham Md Yusoff, Mohamad Helmi Bin Hamid, Norlelawati Mohamad, Karbela Reza Ramlan, Nik Kamarul Arif Nik Kamaruzman, Aliff Omar, Zhafir Asikin, M. Azwan Aziz

**Affiliations:** ^1^ Department of Orthopaedics and Traumatology, Faculty of Medicine Universiti Kebangsaan Malaysia Kuala Lumpur Malaysia

**Keywords:** allografts, anterior cruciate ligament reconstruction, autografts, internal brace, knee

## Abstract

**Background:**

Anterior cruciate ligament reconstruction (ACLR) remains a cornerstone of orthopedic sports medicine, yet graft failure rates are high despite advances in surgical techniques. The Modified UKM Internal Bracing (MUIB) technique combines all‐inside ACLR with internal suture augmentation, potentially offering enhanced graft protection and improved outcomes.

**Methods:**

This retrospective cohort study evaluated 63 patients who underwent isolated primary all‐inside ACLR using the MUIB technique (19 allograft, 44 autograft) between 2020 and 2024. Outcome measures included International Knee Documentation Committee (IKDC) scores, Visual Analog Scale (VAS) pain scores, graft failure rates, Single Leg Hop Test (SLHT) performance, and complication profiles at 3, 6, 12, and 24 months postoperatively.

**Results:**

Both allograft and autograft groups demonstrated significant improvement from preoperative to 2‐year follow‐up (*p* < 0.001). There were no statistically significant differences observed between allograft and autograft groups across all outcome measures, suggesting that the MUIB technique may yield comparable outcomes regardless of graft type. Complete pain resolution (VAS 0) was achieved by 6 months in both groups. Notably, no graft re‐tears were documented throughout the 2‐year follow‐up period, with 95.2% of patients achieving SLHT > 90% at 12 months. No infections, arthrofibrosis, or hardware‐related complications requiring reoperation were recorded.

**Conclusion:**

The MUIB all‐inside ACLR technique yielded excellent 2‐year outcomes in this cohort, characterized by a zero graft failure rate, complete pain resolution, and exceptional functional recovery. However, given the retrospective, uncontrolled design, these findings should be interpreted as preliminary and hypothesis‐generating. Prospective comparative studies are necessary to establish the efficacy and potential superiority of the MUIB technique relative to conventional ACLR approaches.

**Level of Evidence:**

3.

## Graphical abstract

This registry study demonstrates that all‐inside ACL reconstruction using the Modified UKM Internal Bracing (MUIB) technique yields excellent 2‐year functional recovery with zero graft failures or major surgical complications across both autograft and allograft cohorts. These promising preliminary findings warrant confirmation through larger prospective comparative trials.

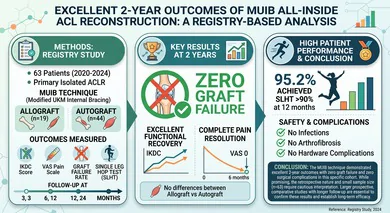

## 1 Introduction

Anterior cruciate ligament (ACL) rupture represents one of the most prevalent and functionally debilitating knee injuries, particularly affecting young, active populations [1]. ACL reconstruction (ACLR) has become a cornerstone of orthopedic sports medicine, with over 200,000 procedures performed annually in the United States alone [2]. Despite advances in surgical techniques and rehabilitation protocols, graft failure remains a significant concern, affecting between 1.8% and 16.7% of patients depending on follow‐up duration and population characteristics [2]. The consequences of failed reconstruction are substantial, often necessitating revision surgery, which consistently yields inferior outcomes compared to primary procedures, with success rates averaging only 70%–75% [2, 3].

The evolution of ACLR techniques has been driven by the pursuit of improved biomechanical stability, faster rehabilitation, and reduced failure rates. The all‐inside technique represents a significant advancement, utilizing retrograde drilling to create sockets rather than full tunnels, thereby preserving bone stock and potentially reducing postoperative pain [4]. Comparative studies have demonstrated that the all‐inside technique offers superior early functional outcomes, with significantly lower Visual Analog Scale (VAS) pain scores and earlier recovery compared to traditional transportal techniques, while maintaining comparable International Knee Documentation Committee (IKDC) and Lysholm scores at intermediate follow‐up [4].

Internal bracing augmentation has emerged as a promising adjunct to ACLR, designed to protect the graft during the critical revascularization and remodeling phase when graft strength is compromised [5, 6]. Biomechanical evidence supports this approach: studies have demonstrated that internal brace augmentation significantly reduces cyclic displacement and increases ultimate failure load compared to fixation alone [5]. Despite the promising biomechanical evidence for the MUIB technique and the established benefits of all‐inside ACLR, there remains a critical gap in the literature regarding clinical outcomes of this combined approach. While cadaveric and biomechanical studies suggest superior graft protection and accelerated rehabilitation potential, the clinical translation of these biomechanical advantages into meaningful patient outcomes, particularly in terms of graft failure rates, functional recovery, and return to sport (RTS), has not been rigorously evaluated.

The problem is further underscored by the multifactorial nature of ACLR failure. Technical errors, particularly tunnel malposition, account for 60%–70% of failures, while traumatic (15%–25%) and biological (10%–15%) causes contribute substantially [2]. The all‐inside approach, with its reliance on precise socket placement, presents a steep learning curve and technical challenges, including the risk of graft bottoming out and inadequate tension [4]. The addition of internal bracing, while biomechanically advantageous, introduces further technical complexity and cost considerations. Given that failure rates in primary ACLR range from 5% to 10% and may reach 35% in high‐risk adolescent populations, establishing the clinical efficacy of this combined technique is imperative.

This study aims to address this knowledge gap by evaluating the 2‐year outcomes of the MUIB all‐inside ACLR technique in a single‐center cohort. We will assess functional outcomes using IKDC score, objective knee stability (Lachman and range of motion), graft failure rates, and complication profiles.

## 2 Methods

### 2.1 Study Design and Setting

This was a retrospective cohort study conducted at a single tertiary referral centre. Data were extracted from a prospectively maintained ACL registry at the institution. The study was conducted in accordance with the Declaration of Helsinki and received UKM medical ethic committee board approval (JEPUKM‐2025‐304). Informed consent was taken during the study.

Data were collected from the ACL registry between May 1, 2025 and May 31, 2026, encompassing patients who underwent ACLR from 2020 to 2024. The following inclusion criteria were applied: (i) isolated primary ACLR, (ii) all‐inside technique of ACLR, and (iii) use of MUIB graft preparation technique. Exclusion criteria were: (i) concomitant meniscal tear requiring repair or meniscectomy, (ii) concomitant chondral procedure (chondroplasty), (iii) multiligament knee reconstruction, and (iv) revision ACLR. Restricting the cohort to isolated all‐inside allograft ACLRs allows for a more focused evaluation of the MUIB technique's efficacy without the confounding influence of additional intra‐articular pathology or surgical procedures.

### 2.2 Surgical Technique

Graft type (allograft vs. autograft) was determined by patient preference and surgeon recommendation following shared decision‐making. Allografts were non‐irradiated, fresh‐frozen peroneus longus tendons procured from a tissue bank. Autografts consisted of quadrupled hamstring tendon grafts harvested using standard techniques. Graft preparation was performed on a GraftPro station (Arthrex) as in Figure 1. Graft length was height‐dependent: 62 (< 155 cm), 65 (155–179 cm), or 67 mm (> 179 cm), with corresponding internal bracing adjustments. The femoral TightRope II and tibial ABS button were loaded, while trifold FiberTape loops (2 mm) were secured with #0 FiberWire, avoiding interference with the TightRope to preserve gliding. The graft was quadruple‐folded over the loops, whipstitched with Ethibond, and compacted to target length. Stump‐end stitches and two additional reinforcing stitches secure the construct to maximize Sharpey fiber formation, and the graft was marked for socket lengths.

**FIGURE 1:**
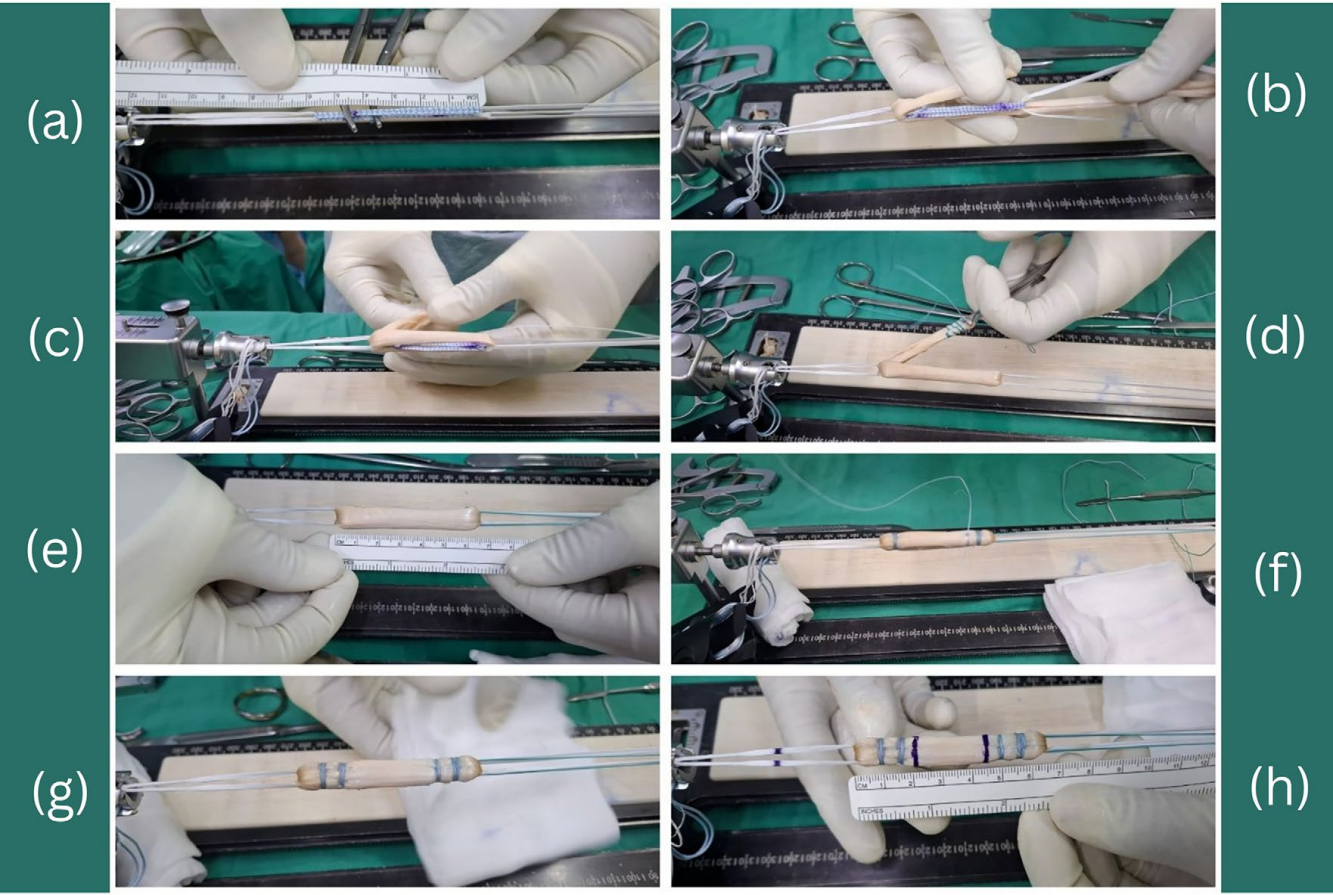
Graft preparation. (a) First, the ACL TightRope implants (both the TightRope II RT and the Attachable Button System) were looped one and a half times around the GraftPro base, and a trifolded FiberTape loop was positioned alongside them. The FiberTape was then fixed in place using a No. 0 FiberWire suture and trimmed to a length of 58 mm. (b, c) Next, the graft was configured into a quadruple‐folded construct to ensure even load sharing, with the FiberTape incorporated internally. (d–f) Both terminal ends of the graft were whipstitched using 2.0 Ethibond sutures and (g, h) bringing the overall graft length to 65 mm. Finally, the GraftLink loop technique was employed, and an additional pair of reinforcing sutures was applied to both sides of the graft to enhance construct stability.

For the all‐inside technique, the graft was passed into the femoral socket and the TightRope RT was deployed at the lateral cortex. Sequential tightening seats the graft. Tibial fixation was achieved by delivering the ABS button with the graft and FiberTape ends into the tibial tunnel. Primary fixation was performed at 0° extension (or negative zero for hyperlaxity). After cycling the knee, final tightening occurs in full hyperextension, with supplementary sutures tied to the ABS.

Postoperatively, arthrogenic muscle inhibition was minimized from Day 1 via multimodal analgesia, cryotherapy/compression, and graded range‐of‐motion exercises. Protected weight‐bearing is maintained for 6 weeks, followed by progressive ROM restoration (prioritizing full extension), weight‐bearing advancement guided by quadriceps control, and closed‐chain strengthening.

### 2.3 Data Collection

Data were extracted from the institutional ACL registry, which prospectively records preoperative, perioperative, and postoperative data for all ACLR patients. The following demographic and clinical data were collected: (i) demographic variables: age, sex, body mass index (BMI), and activity level, (ii) clinical examination: preoperative and postoperative knee examination findings, including range of motion, ligamentous laxity using Lachman test, (iii) patient‐reported outcomes using IKDC [7] at 3 month, 6 month, 12 month, and 2 years, (iv) RTS readiness using Single Leg Hop Test (SLHT) was performed at 9 and 12 months postoperatively to assess functional performance, and (v) graft re‐tear defined as symptomatic graft re‐tear confirmed by imaging and requiring revision surgery, assessed at minimum 2‐year follow‐up or other complications. Graft failure was defined as symptomatic graft re‐tear confirmed on MRI or revision surgery, and was ascertained through clinical evaluation, patient self‐report, and imaging when clinically indicated. There were no missing data recorded in all patients and no loss of follow up.

### 2.4 Statistical Analysis

Continuous variables (age, body mass index) were presented as mean ± standard deviation, preoperative Tegner score was expressed as median with interquartile range, and categorical variables (gender, mechanism of injury and dominant leg injured) were expressed as frequencies and percentages. IKDC scores were summarized as mean and standard deviation at each assessment. Longitudinal outcomes were analyzed using a repeated‐measures linear model fitted through the MIXED procedure. The model included assessment time (preoperative, 12, 24, 52, and 104 weeks), graft type (allograft or autograft), and their interaction as fixed effects. Time was treated as a categorical variable to allow a nonlinear recovery pattern. An unstructured within‐patient covariance matrix accounted for correlations between repeated measurements and permitted different variances across assessments. Parameters were estimated using restricted maximum likelihood, without a separate patient random intercept. Type III *F* tests assessed the effects of time, graft type and the time × graft‐type interaction. The interaction tested whether IKDC trajectories differed between graft groups. Estimated marginal means and between‐group mean differences were reported with pointwise 95% confidence intervals. Visit‐specific between‐group comparisons were evaluated using model‐based contrasts, with Holm adjustment applied across the five comparisons. The model was unadjusted for clinical covariates. All tests were two‐sided, and adjusted *p* values below 0.05 were considered statistically significant. For the preoperative‐to‐postoperative change in VAS scores, the Wilcoxon signed‐rank test was used for within‐group comparisons, while the Mann–Whitney *U* test was applied for between‐group comparisons, as the data were not normally distributed. All statistical analyses were performed using SPSS software Version 29 (IBM Corp., Armonk, NY, USA).

## 3 Results

### 3.1 Demographic

Over 387 arthroscopic procedures were performed, from 2020 to 2024, 215 were ACLR surgery. A total of 63 patients underwent isolated all‐inside ACLR during the study period, comprising 19 allograft recipients and 44 autograft recipients. The mean age of the entire cohort was 34.9 ± 8.2 years, with no significant difference between allograft (34 ± 8.47 years) and autograft (35 ± 8.18 years) groups (*p* = 0.514). The cohort was predominantly male (85.7%, *n* = 54), with females accounting for 14.3% (*n* = 9). Non dominant‐sided ACL tears were more prevalent (68.3%, *n* = 43) compared to dominant‐sided injuries (31.7%, *n* = 20). Sports‐related injuries constituted the predominant mechanism (93.7%, *n* = 59), with only 6.3% (*n* = 4) attributed to non‐sporting trauma (Table 1).

**TABLE 1:** Baseline demographic of 63 patients who underwent isolated all inside ACL reconstruction using MUIB graft.

	Total	Allograft (19)	Autograft (44)	*p*
Age	34.9 (8.2)^a^	34 (8.47)^c^	35 (8.18)^c^	0.514
Anthropometric assessment				
Weight	77.1 (8.8)^a^	74 (14)^c^	77 (12)^c^	0.471
Height	172.6 (7.09)^a^	175 (8)^c^	174 (5)^c^	0.958
Body mass index	25.8 (1.72)^a^	26.1 (1.72)^c^	26.1 (1.74)^c^	0.610
Gender				
Female	9 (14.3)^b^	3 (33.3)^d^	6 (66.7)^d^	0.298
Male	54 (85.7)^b^	16 (29.6)^d^	38 (70.4)^d^	
Laterality of injury				
Dominant leg ACL tear	20 (31.7)^b^	16 (37.2)^d^	27 (62.8)^d^	0.074
Non dominant leg ACL tear	43 (68.3)^b^	3 (15)^d^	17 (85)^d^	
Mechanism of injury				
Sports related injury	59 (93.7)^b^	18 (30.5)^d^	41 (69.5)^d^	0.816
Trauma	4 (6.3)^b^	1 (25)^d^	3 (75)^d^	

^a^ Age and anthropometric assessment were expressed as mean and standard deviation.

^b^ Gender, laterality of injury, and mechanism of injury were expressed as frequency and percentages.

^c^ Age and anthropometric assessment were expressed as median and IQR and compared using Mann Whitney test, and *p* < 0.05 was considered significant.

^d^ Gender, laterality of injury, and mechanism of injury were compared using chi square test, and *p* < 0.05 was considered significant.

### 3.2 Post Operative outcomes

Following an early postoperative decrease (Figure 2), IKDC scores (Table 2) exhibited a subsequent recovery over the follow‐up period (allograft [88.53 ± 3.34] vs. autograft [87.82 ± 3.04] groups). Although temporal fluctuations in scores were statistically significant, the trajectory of recovery did not differ appreciably between allograft and autograft cohorts. Pairwise comparisons at individual time points yielded no significant differences following Holm correction for multiple testing. Accordingly, these results do not provide evidence of equivalence between the two graft types. At the final 2‐year follow‐up, both groups achieved excellent IKDC scores of 98.5 ± 4.5, with allograft and autograft groups both reaching a ceiling score of (allograft [97.42 ± 6.18] vs. autograft [99.07 ± 3.53]) (*p* = 0.740). The overall improvement from preoperative to 2‐year follow‐up was highly significant (*p* < 0.001) (Table 3). The overall improvement from preoperative to 2‐year follow‐up was 10.54 points (95% CI: 9.24–11.84; *p* < 0.001). The allograft group demonstrated an improvement of 8.89 points (95% CI: 5.56–12.23; *p* < 0.001), while the autograft group improved by 11.25 points (95% CI: 10.01–12.49; *p* < 0.001). The between‐group difference in improvement was −2.36 points (95% CI: −5.15 to 0.44; *p* = 0.097).

**FIGURE 2:**
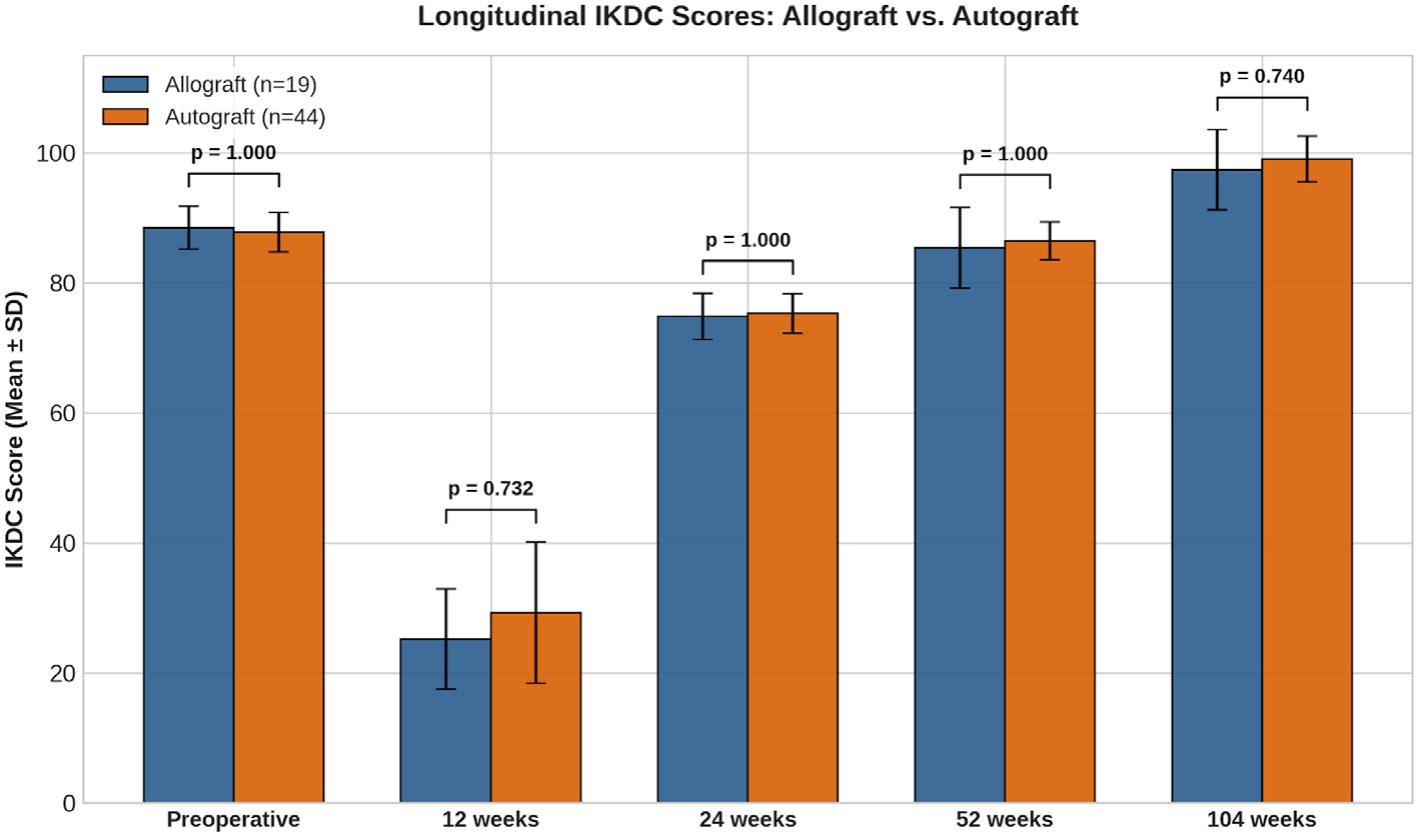
Longitudinal comparison of IKDC scores between allograft and autograft cohorts over a 2‐year follow‐up. Bars display mean International Knee Documentation Committee (IKDC) functional scores for allograft (*n* = 19) and autograft (*n* = 44) groups preoperatively and at 12, 24, 52, and 104 weeks postoperatively. Error bars represent standard deviations (*p* value < 0.05 is considered significant). Both groups experienced an initial postoperative decline at 12 weeks followed by sustained recovery through 104 weeks. *p* values indicate visit‐specific between‐group comparisons following Holm adjustment derived from a repeated‐measures linear mixed model. No statistically significant differences were observed between graft types at any evaluation point (*p* > 0.05).

**TABLE 2:** Longitudinal International Knee Documentation Committee (IKDC) scores and between‐group comparisons over 2‐year follow‐up.

Assessment	Overall, mean ± SD	Allograft, mean ± SD	Autograft, mean ± SD	Allograft – autograft difference (95% CI)	Holm‐adjusted *p*
Preoperative	88.03 ± 3.12	88.53 ± 3.34	87.82 ± 3.04	0.71 (−1.01 to 2.43)	1.000
12 weeks	28.10 ± 10.14	25.26 ± 7.72	29.32 ± 10.87	−4.06 (−9.57 to 1.46)	0.732
24 weeks	75.21 ± 3.20	74.89 ± 3.57	75.34 ± 3.06	−0.45 (−2.22 to 1.32)	1.000
52 weeks	86.16 ± 4.17	85.42 ± 6.23	86.48 ± 2.92	−1.06 (−3.35 to 1.24)	1.000
104 weeks	98.57 ± 4.50	97.42 ± 6.18	99.07 ± 3.53	−1.65 (−4.10 to 0.81)	0.740

*Note:* Values are presented as mean ± standard deviation. Between‐group differences (allograft − autograft) are presented with 95% confidence intervals. *p* values were derived from model‐based contrasts within a repeated‐measures linear mixed model, with Holm adjustment applied across the five visit‐specific comparisons to control the family‐wise error rate. A statistically significant time effect was observed (*p* < 0.001), indicating substantial improvement in IKDC scores over the follow‐up period. However, neither the main effect of graft type nor the time‐by‐graft‐type interaction reached statistical significance, suggesting that the trajectory of recovery did not differ between allograft and autograft recipients. No visit‐specific between‐group comparison was statistically significant after Holm adjustment.

**TABLE 3:** Change in International Knee Documentation Committee (IKDC) scores from preoperative assessment to 104 weeks postoperatively, stratified by graft type.

Population or comparison	*N*	Estimated change (95% CI), points	*p*
Overall cohort	63	10.54 (9.24 to 11.84)	< 0.001
Allograft	19	8.89 (5.56 to 12.23)	< 0.001
Autograft	44	11.25 (10.01 to 12.49)	< 0.001
Between‐group difference in change: allograft − autograft	63	−2.36 (−5.15 to 0.44)	0.097

*Note:* Data are presented as estimated mean change in IKDC score (points) with 95% confidence intervals. Changes were derived from a repeated‐measures linear mixed model, with the preoperative assessment serving as the reference time point. *p* values are based on model‐based contrasts. Both allograft and autograft groups demonstrated significant improvements from preoperative to 2‐year follow‐up (*p* < 0.001 for both). Although the estimated improvement was numerically larger in the autograft group (11.25 points) than in the allograft group (8.89 points), the between‐group difference in change was not statistically significant (difference: −2.36 points; 95% CI: −5.15 to 0.44; *p* = 0.097), indicating that the magnitude of improvement did not differ significantly between graft types.

Preoperative VAS pain scores (Figure 3) were low across both groups (total: 3.1 ± 0.7; allograft: 3 ± 1; autograft: 3 ± 0; *p* = 0.06). At 3 months postoperatively, a significant reduction in pain was observed, with autograft recipients reporting superior pain control (1 ± 1) compared to allograft recipients (2 ± 2) (*p* = 0.003). By 6 months, both groups achieved complete resolution of pain, with median VAS scores of 0 (IQR: 0) in both groups. 84.1% (*n* = 53) managed to perform the SLHT and achieved a score > 90% at 9 months, and 95.2% (*n* = 60) achieved a score > 90% at 12 months.

**FIGURE 3:**
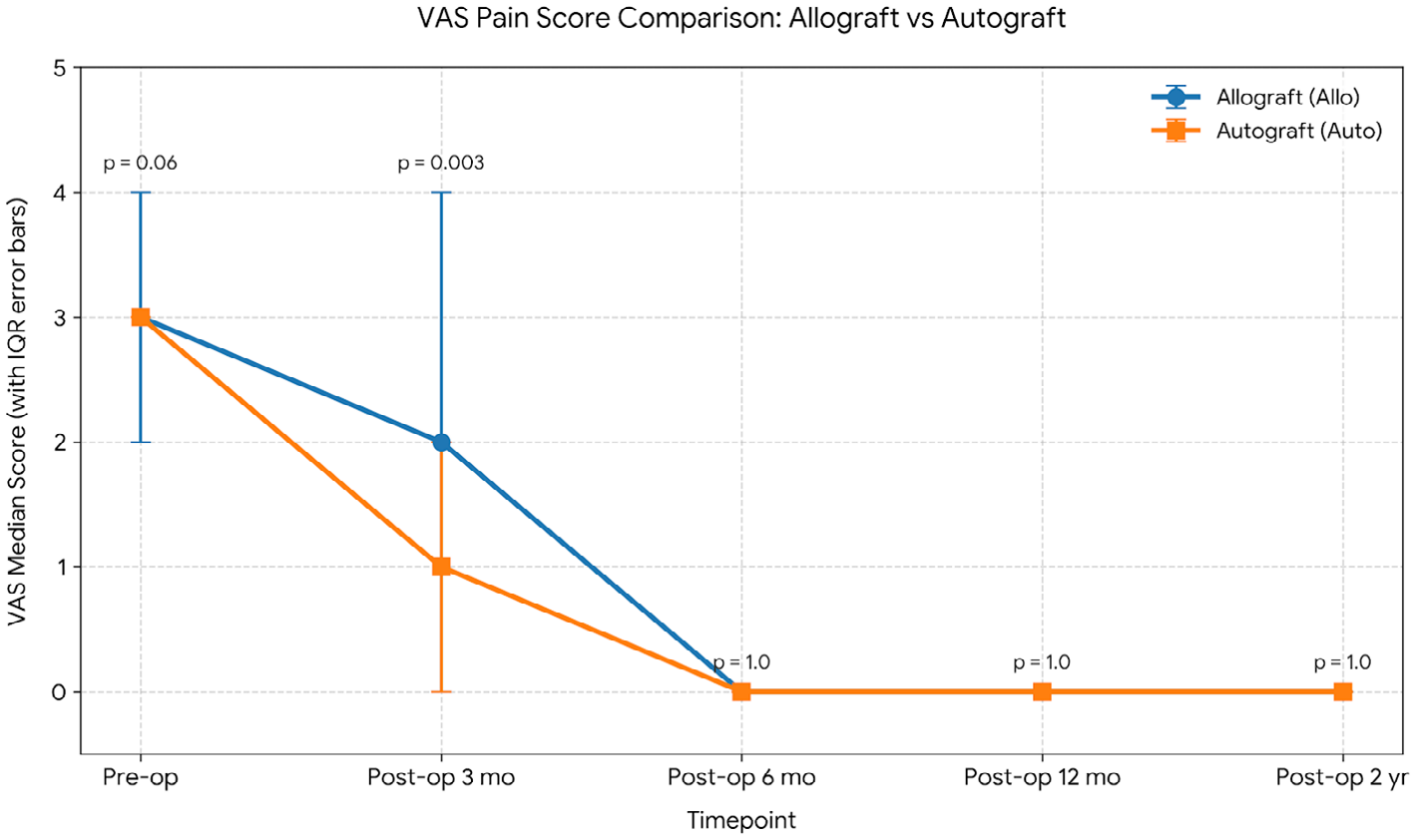
Comparison of VAS score at preoperative, 3‐month postoperative, 6‐month postoperative, 12‐month postoperative, and 2‐year postoperative, between allograft and autograft using Mann–Whitney test.

In terms of postoperative laxity, 11.1% (*n* = 7) has Grade 0 Lachmann test, and 88.9% (*n* = 56) had Grade 1 Lachmann test. All patients had full range of motion. There were no instability reported, and no graft re‐tears were documented throughout the 2‐year follow‐up period in either group (95% CI: 0%–5.7%). There were no cases of infection, arthrofibrosis, or hardware‐related complications requiring reoperation recorded.

## 4 Discussion

This study represents the first clinical evaluation of the Modified UKM Internal Bracing (MUIB) all‐inside ACLR technique with a minimum 2‐year follow‐up. Our findings demonstrate that the MUIB technique, when applied to isolated all‐inside ACLR using either allograft or autograft, yields excellent functional outcomes, complete pain resolution by 6 months, and, most notably, a 0% graft failure rate at 2 years. The MUIB technique represents a paradigm shift in graft preparation, embedding a trifold FiberTape internal brace within the graft core rather than fixing it independently. This design addresses a critical limitation of conventional internal bracing, the “windshield‐wiper” motion that can lead to tunnel widening and subsequent graft failure [8].

### 4.1 Graft failure rate

The observed zero graft failure rate, while encouraging, should be interpreted with caution. With 63 patients, the upper bound of the 95% confidence interval extends to 5.7%, indicating that the true failure rate could be as high as approximately 6% in the broader population. This finding provides preliminary evidence of safety but does not establish that the MUIB technique eliminates the risk of graft failure entirely. This rate is substantially lower than the 4.5% 2‐year failure rate reported in the Multicenter Orthopaedic Outcomes Network (MOON) cohort of 2497 patients [9]. Our 0% failure rate falls at the extreme lower end of the spectrum; autograft failure rates in the literature range from 0% to 9.4%, while allograft failure rates range from 0% to 26.5% [10]. The protective effect of the MUIB technique is consistent with the growing body of evidence supporting suture augmentation in ACLR. A systematic review and meta‐analysis of suture augmentation demonstrated a 62% relative risk reduction in graft failure (RR 0.38; 95% CI 0.19–0.73; *p* = 0.004) [11]. This effect was particularly pronounced in younger patients (< 20 years). Our finding of zero failure in both allograft and autograft groups suggests that the MUIB technique, with its unique trifold internal bracing configuration embedded within the graft, may confer even greater protection than conventional suture augmentation. Importantly, our results align with a recent study of ACLR with internal bracing using quadrupled hamstring autograft, which also reported no graft failures requiring revision surgery at 2 years [12]. The authors concluded that ACLR with internal bracing is a safe and secure surgical technique. It is important to note that the biomechanical evidence provides mechanistic support for internal bracing but does not substitute for clinical outcome data. Furthermore, the systematic reviews and meta‐analyses cited [8, 10] include populations with different demographic characteristics, and extrapolation of these findings to our cohort comprising predominantly male, middle‐aged patients should be undertaken with caution [11].

### 4.2 Post operative clinical outcomes

The most interesting finding of this study is the absence of significant differences between allograft and autograft groups across all outcome measures, including IKDC scores (both reaching near 100 at 2 years), VAS pain scores (both 0 at 6 months), and graft failure rate (0% in both groups). It is important to emphasize that the absence of statistically significant differences between allograft and autograft groups does not constitute evidence of equivalence. The small allograft subgroup (*n* = 19) and the absence of a predefined equivalence margin or non‐inferiority design preclude such a conclusion. At best, these findings suggest that allograft outcomes in the context of the MUIB technique are comparable to autograft outcomes within the limits of this cohort, and that further investigation with adequate statistical power is warranted. A systematic review and meta‐analysis of patients 19 years and younger reported pooled failure rates of 8.5% for bone‐patellar tendon‐bone autografts, 16.6% for hamstring autografts, and 25.5% for allografts, with allografts being significantly more likely to fail (odds ratio 3.87; 95% CI 2.24–6.69) [13]. Similarly, another systematic review reported that hamstring and tibialis posterior allografts demonstrate failure rates of 6.2%–13.8%, particularly in younger patients [9, 10]. However, our findings are not without precedent. An updated systematic review comparing autograft versus nonirradiated allograft found that the two graft types demonstrate similar patient‐reported outcomes and graft failure rates. The authors reported that graft failure ranged from 0% to 9.4% in the autograft group and 0%–26.5% in the allograft group, with no significant differences in objective IKDC scores, anteroposterior laxity, or patient‐reported outcomes between groups [10].

Our study contributes to this evolving narrative by demonstrating that when allografts are protected by the MUIB internal bracing construct, they can achieve outcomes equivalent to, and in this cohort, indistinguishable from autografts. The equivalence we observed suggests that the MUIB technique may mitigate the inherent biological disadvantages of allograft tissue. By embedding the FiberTape internally, the MUIB construct provides immediate load‐sharing that protects the allograft during the prolonged incorporation phase, the period when allografts are most vulnerable to failure. The internal brace may compensate for the slower graft ligamentization and reduced cellular infiltration characteristic of allografts, effectively bridging the gap until the graft achieves sufficient biological strength.

The IKDC scores in our cohort improved from a preoperative mean of 88–90 to 98.5 at 2 years, with both allograft and autograft groups reaching a ceiling score of near 100. These scores compare favorably with published benchmarks. A prospective randomized trial comparing all‐inside and complete tibial tunnel techniques reported mean 1‐year IKDC scores of 86 ± 11 in the all‐inside group and 88 ± 14 in the complete tibial tunnel group, with no significant difference between techniques [14]. Our 12‐month IKDC of 86.1 is comparable, and our 2‐year score of 98.5 represents continued improvement beyond the 1‐year mark.

A recent systematic review and meta‐analysis of 12 randomized controlled trials found that all‐inside ACLR potentially promotes superior mid‐to‐long‐term subjective outcomes (mean difference in IKDC: 2.94; 95% CI 0.53–5.35; *p* = 0.02) [15]. However, the same meta‐analysis raised concerns about increased knee laxity (mean difference 0.71; 95% CI 0.24–1.19; *p* = 0.003) and a potential trend toward increased re‐rupture risk (OR 2.25; 95% CI 0.76–6.65; *p* = 0.14) with the all‐inside technique [15]. Our study did not assess objective laxity measures such as KT‐1000 arthrometer measurements, which limits our ability to comment on this potential concern. However, the Lachmann test and zero failure rate in our cohort suggests that any residual laxity, if present did not translate into clinical failure.

Importantly, the all‐inside approach has been associated with reduced postoperative pain and a diminished early inflammatory response [16]. This is consistent with our finding of complete pain resolution (VAS 0) by 6 months in both groups, with autograft recipients demonstrating superior pain control at 3 months (VAS 1 ± 1 vs. 2 ± 2, *p* = 0.003).

At 9 months postoperatively, 84.1% of patients achieved a SLHT limb symmetry index > 90%, increasing to 95.2% at 12 months. These rates compare favorably with the literature. A prospective longitudinal study reported that the percentages of patients passing all RTS criteria were low at both 6 and 9 months after ACLR [17]. In this study, at 6 months, only two patients (3.2%) passed all RTS criteria, increasing modestly to seven patients (11.3%) at 9 months. Alarmingly, nearly half the cohort (46.8%, *n* = 29) failed to meet the strength criterion at 60°/s at 9 months, representing a significant barrier to safe RTS. Furthermore, IKDC scores did not show corresponding improvement over time, suggesting that functional recovery lags behind patient‐reported outcomes. The meta‐analysis found that suture augmentation was associated with a significantly higher RTS rate compared with standard ACLR (RR 1.12; 95% CI 1.00–1.24; *p* = 0.04) [11]. The SLHT represents a single component of a comprehensive RTS assessment. While our findings indicate that 95.2% of patients achieved a limb symmetry index > 90% at 12 months, this should not be interpreted as evidence of RTS readiness. Future research should incorporate validated RTS criteria to evaluate the functional benefits of the MUIB technique more comprehensively.

The absence of infections, arthrofibrosis, or hardware‐related complications in our cohort confirms the safety of the MUIB technique. This is consistent with the finding that suture augmentation is not associated with increased reoperation or complication rates. A recent study of ACLR with internal bracing similarly reported no complications such as postoperative joint stiffness, synovitis, infection, or graft failure.

## 5 Limitations

Several limitations warrant acknowledgement. First, the absence of a conventional ACLR control group represents a significant limitation of this study. Consequently, our findings cannot be interpreted as evidence of superiority of the MUIB technique over standard ACLR approaches. Rather, the observed outcomes, particularly the zero graft failure rate, provide preliminary evidence that the MUIB technique is safe and effective in this cohort and warrants further investigation through rigorously designed comparative studies. Second, the sample size (*n* = 63) limits subgroup analyses and generalisability. Third, the allograft group (*n* = 19) was smaller than the autograft group (*n* = 44), though baseline characteristics were well‐matched. Fourth, the 2‐year follow‐up, while adequate for assessing early graft failure, is insufficient to evaluate longer‐term outcomes such as osteoarthritis development. Fifth, the MUIB technique was performed by a single surgeon at a high‐volume centre, raising the possibility of a learning‐curve effect that may limit generalisability to lower‐volume settings. Finally, the absence of objective stability measurements (KT‐1000 or KT‐2000) limits the conclusion on residual laxity, which is a concern raised by recent meta‐analyses of all‐inside techniques.

Further research with larger sample sizes, longer follow‐up, and randomized controlled designs is warranted to confirm these findings. Comparative studies with nonaugmented ACLR and conventional internal bracing techniques would help establish the relative efficacy of the MUIB approach. Long‐term follow‐up is essential to evaluate whether the biomechanical advantages translate into reduced osteoarthritis risk.

## 6 Conclusion

In conclusion, this single‐centre observational study demonstrates that the MUIB all‐inside ACLR technique is associated with excellent 2‐year outcomes in a cohort of 63 patients, with no graft failures, complete pain resolution by 6 months, and IKDC scores approaching 100. Although no significant differences were observed between allograft and autograft groups, the study was not designed or powered to establish equivalence. The results should be interpreted as preliminary and hypothesis generating, providing a foundation for future prospective randomized trials to determine the comparative effectiveness and potential superiority of the MUIB technique.

## Author Contributions

**Badrul Akmal Hisham Md Yusoff:** conceptualization, investigation, writing – original draft, writing – review and editing, methodology, formal analysis, supervision. **Mohamad Helmi Bin Hamid:** conceptualization, investigation, writing – original draft, writing – review and editing, methodology, formal analysis. **Norlelawati Mohamad:** conceptualization, investigation, writing – original draft, writing – review and editing. **Karbela Reza Ramlan:** methodology, writing – review and editing, writing – original draft. **Nik Kamarul Arif Nik Kamaruzman:** conceptualization, investigation, writing – review and editing, writing – original draft, methodology. **Aliff Omar:** conceptualization, methodology, writing – original draft, writing – review and editing, investigation. **Zhafir Asikin:** conceptualization, investigation, writing – original draft, writing – review and editing. **M. Azwan Aziz:** conceptualization, investigation, writing – original draft, writing – review and editing, methodology, formal analysis, supervision.

## Funding

The authors have nothing to report.

## Ethics Statement

This study has been approved by the UKM Medical Ethic Committee JEP 2025 304.

## Conflicts of Interest

The authors declare no conflicts of interest.

## Data Availability

The data that support the findings of this study are openly available in Mendeley Data at https://data.mendeley.com, reference number 10.17632/42b58sg6dg.1.
